# Gas Flow Models and Computationally Efficient Methods for Energy Network Optimization

**DOI:** 10.1021/acs.iecr.3c04308

**Published:** 2024-03-19

**Authors:** Behnam Akbari, Paolo Gabrielli, Giovanni Sansavini

**Affiliations:** Institute of Energy and Process Engineering, ETH Zurich, 8092 Zurich, Switzerland

## Abstract

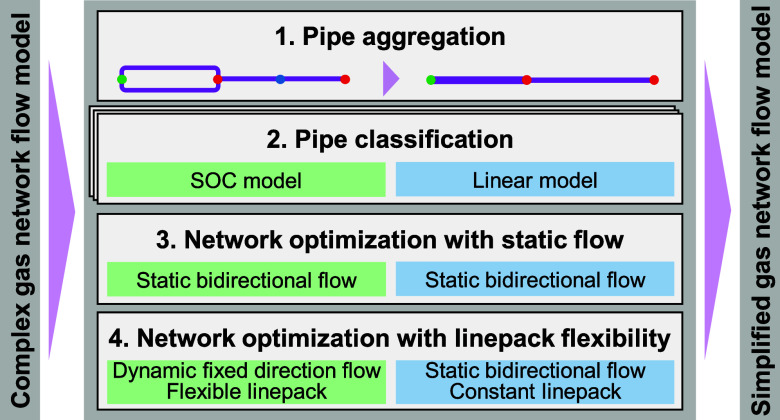

The equations governing gas flow dynamics are computationally challenging for energy network optimization. This paper proposes an efficient solution procedure to enable tractability for an hourly resolved yearly decision horizon. The solution procedure deploys linear and second-order cone gas flow models alternatively based on the length–diameter ratio of pipes, achieving maximum efficiency within accuracy limits. Moreover, it addresses the computational complexity of bidirectional pipe flows by fixing the associated integer variables according to a preceding optimization with a static flow approximation. The procedure also precisely aggregates parallel and serial pipes for increased efficiency. Mathematical derivations and single-pipe analyses substantiate the model selection criterion. Network optimizations validate the accuracy, success rate, and scalability of the procedure, achieving up to 3.1% cost savings compared to static models, enhancing the success rate by a minimum of 96%, and boosting computational efficiency up to 3 orders of magnitude over full dynamic models.

## 1 Introduction

Mathematical optimization has emerged as an effective tool for the expansion and operation planning of energy networks involving gaseous fuels, such as natural gas and hydrogen. In this context, various optimization problems have been studied, including gas quality satisfaction and linepack management in natural gas network operations.^1^ Recently, these optimization problems have found new applications in studying hydrogen injection into natural gas networks^2^ and the operation of dedicated hydrogen networks.^3^ The research has also extended to the expansion planning of integrated energy networks, covering power, natural gas, and hydrogen carriers.^4,5^

A major challenge in these optimization problems is the nonconvex nature of the equations governing the gas flow dynamics in network pipes. Thus, techniques such as semidefinite relaxation,^6,7^ second-order cone (SOC) relaxation,^3,8,9^ and linear approximation^10−15^ have been deployed to obtain computationally tractable dynamic flow models for operations optimization on daily horizons.

In the context of expansion planning optimization, gas flow models have been further simplified. Static models^5,16−20^ neglect linepack flexibility and assume immediate gas delivery, while transport models^21−24^ also disregard pressure–flow relations. Neglecting linepack flexibility can result in overestimating costs, and disregarding pressure–flow relations can lead to demand shedding. Although metaheuristic^25,26^ and simulation^27,28^ models capture linepack flexibility, they are prone to suboptimality and rely on predefined boundary conditions (e.g., compression ratios and boundary pressures). Additionally, these models have been applied with diverse temporal resolutions, with static models considering merely 1–20 snapshots, transport and metaheuristic models capturing hourly operations, and simulation models representing subhourly dynamics.

Therefore, a research gap exists in integrating linepack flexibility into deterministic optimization methods for energy network expansion planning.^4^ This paper aims to fill this gap by proposing a solution procedure suitable for an hourly resolved annual time horizon, which is the benchmark temporal resolution in energy system expansion planning.^29^ Moreover, it offers decision support for selecting gas flow models by quantifying the trade-offs between computational efficiency and model accuracy. The proposed solution procedure builds on three novel modeling techniques:

1. **Pipe aggregation:** This technique reduces the problem size by aggregating parallel and serial pipes while preserving pressure–flow relations and total linepack. Mathematical derivations confirm the steady-state and transient precision of parallel aggregation and the steady-state precision of serial aggregation. Computational experiments demonstrate the transient accuracy of serial pipe aggregation.
2. **Pipe classification:** Network pipes are classified for representation with linear and SOC gas flow models, optimizing for computational efficiency within accuracy limits. Notably, the proposed linear model improves the steady-state accuracy of the *short pipe model* in the literature^30^ by including pressure-induced flow bounds.
3. **Fixing flow directions:** The solution procedure models bidirectional pipe flows using integer variables in a static flow optimization and fixes flow directions accordingly to avoid the associated computational complexity in dynamic flow optimization. While fixing flow directions has previously been applied in daily operation optimizations with dynamic flows^9,11^ and planning optimizations without temporal dynamics,^17,18,31^ our approach combines the scalability of static flow models with the accuracy of dynamic ones. This combination enables integrating linepack flexibility into hourly resolved optimizations with an annual time horizon.

This paper is organized as follows. Section 2 develops gas flow models with varying accuracy and computational efficiency. Section 3 presents metrics for assessing the accuracy of the gas flow models. Section 4 employs the gas flow models in a computationally efficient solution procedure for network optimization while ensuring a specified accuracy. Section 5 assesses the gas flow models in single-pipe analyses and network optimizations. Section 6 concludes the article with insights and potential future directions.

## 2 Gas Flow Models

The optimization of gas networks, encompassing natural gas and hydrogen, follows the general form
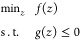
1

Here, the objective function *f*(*z*) may include the cost of gas supply^3^ and network expansion,^16^ and the constraint function *g*(*z*) captures the operation and investment considerations, including the physical and technical constraints of gas flow in network pipes. Section 2 focuses on the derivation of the gas flow constraints, and it starts with a continuous gas flow model based on partial differential equations and, by progressive simplification, obtains discrete dynamic and static models with superior computational efficiency. We do not repeat constraints that remain unchanged in the simplification steps. Instead, we summarize the constraints of the gas flow models in Section 2.6.

### 2.1 Continuous Model

The equations of continuity (eq 2) and momentum (eq 3) govern gas flow dynamics in a pipe under isothermal conditions^10^
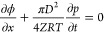
2
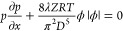
3where *t* and *x* denote time and one-dimensional space; ϕ is the mass flow rate; *p* is the gas pressure; *D* is the pipe’s diameter; and λ, *Z*, *R*, and *T* are the Darcy friction factor, compressibility factor, specific gas constant, and temperature, respectively. Equation 3 relies on the following assumptions

1. The gravitational force is neglected due to a horizontal pipe assumption.
2. The inertial and kinetic forces are neglected as their contribution is merely 1% of the frictional force^32^ and the flow velocities are insignificant compared to the sound’s velocity in the gas.^33^
3. The compressibility factor’s dependence on pressure is neglected as its deviation is below 3% under normal operational pressures.^32^

The pressure bounds are enforced as
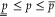
4

The notations · and ·̅ generally represent lower and upper bounds on decision variables, respectively.

Mass flow is restrained to the cross-section capacity as
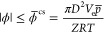
5where *V*_e_ is the velocity limit to avoid pipe erosion, vibration, and noise.^34^

To assess the steady state, we set  in eq 2. This requires , i.e., mass flow is equal along the pipe. Integrating eq 3 over the pipe length *x* ∈ [0,*L*] yields
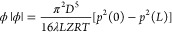
6

We repeat the integration over *x* ∈ [0,*x*_0_], where 0 ≤ *x*_0_ ≤ *L*. Setting the resulting equation for ϕ equal to the one in eq 6 follows that *p*(0) ≤ *p*(*x*_0_) ≤ *p*(*L*), indicating that it suffices to enforce the pressure bounds, eq 4, at the extreme nodes. Plugging the pressure bounds into eq 6 yields a bound on the steady-state mass flow
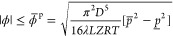
7

The pressure-induced bound  can be expressed in terms of cross-section capacity  as
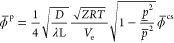
8

### 2.2 Discrete Nonconvex Dynamic Model

**Figure 1:**
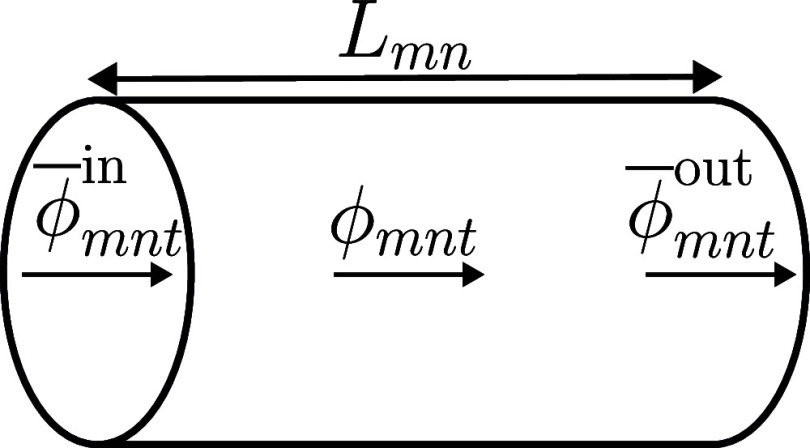
Mass flow variables in a pipe segment.

For integration into energy network optimization, eq 1, we discretize the continuous model in space and time, with steps of *L*_*mn*_ and Δ*t*, using midpoint and backward Euler methods,^35^ respectively. For a pipe segment *mn* (Figure 1), eq 2 is expressed as
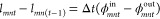
9a
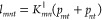
9b
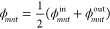
9cand eq 3 is expressed using the Weymouth equation
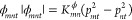
10where
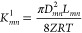
11a
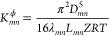
11b

The variable *l*_*mnt*_ represents the line pack, namely, the mass of gas contained in the pipe segment *mn*. The spatiotemporal variations in temperature and, hence, compressibility factor can be readily incorporated as *T*_*mnt*_ and *Z*_*mnt*_, respectively, resulting in time-dependent line pack and flow parameters, i.e., *K*_*mnt*_^l^ and *K*_*mnt*_^ϕ^. Without extra computational complexity, this incorporation captures the positive correlation among the temperature, dynamic speed, and pressure drop.

The pressure bounds are enforced as
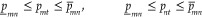
12

The cross-section capacity is enforced as
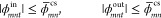
13

### 2.3 Discrete Second-Order Cone Dynamic Model

The nonconvex Weymouth eq 10 is relaxed to an SOC constraint
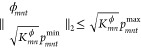
14

As the constraint may not hold with equality, the relaxation gap, ϵ_*mnt*_, quantifies the deviation from eq 10
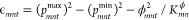
15

To tighten the relaxation gap, we linearize it as γ_*mnt*_ around a point 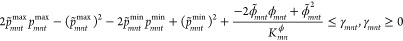
16and augment the objective function, *f*, with the penalty term
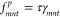
17where τ is the penalty coefficient. A relaxed model (τ = 0) yields a lower bound on *f*, while a penalized model (τ > 0) yields a solution with enhanced feasibility.

The flow direction should match the direction of the pressure drop, which is enforced with the aid of an integer variable, *y*_*mnt*_, and the following linear constraints
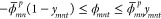
18a
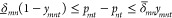
18b
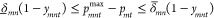
18c
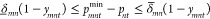
18d
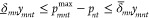
18e
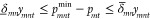
18fwhere  and .

### 2.4 Discrete Second-Order Cone Static Model

For pipes with small volume, *K*_*mn*_^l^ is small, and eq 9a,9b,9c is approximated as
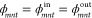
19which removes the linkage between time steps and hence creates a static model.

As the pressure variables (*p*) are absent from eq 19, we reduce the number of nonlinear terms in the Weymouth equation in eq 10 by representing |*p*_*mt*_^2^ – *p*_*nt*_^2^| and *p*_*mt*_^2^ with *β*_*mnt*_ and *β*_*mt*_, respectively. Then, eq 10 is relaxed to SOC and linear constraints
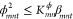
20a
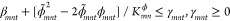
20bwhere γ_*mnt*_ linearly approximates the relaxation gap, ϵ_*mnt*_, around 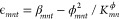
21

Similar to the SOC dynamic model, the objective function is augmented with the penalty term eq 17 to minimize the relaxation gap.

Valid inequalities limit the relaxation gap
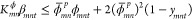
22a
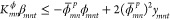
22b

The flow direction should match the direction of the pressure drop, which is enforced by
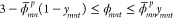
23a
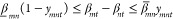
23b
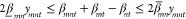
23c
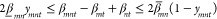
23dwhere .

The pressure bounds are enforced as
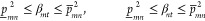
24

### 2.5 Discrete Linear Static Model

For sufficiently short pipes, *K*_*mn*_^ϕ^ is large, and eq 10 is approximated as
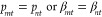
25

The choice between the alternative forms in eq 25 depends on the choice of pressure variables, i.e., *p* or *β*.

The mass flow constraint due to pressure bounds is represented as
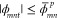
26

### 2.6 Summary of Discrete Gas Flow Models

The mathematical formulations of the gas flow models are summarized in Table 1, while their computational complexities are outlined in Table 2. Specifically, nonconvexity introduces multiple local optima, complicating the search for the global optimum; time-linking constraints hinder problem decomposition into manageable instances; nonlinear constraints necessitate more intensive computations for solution algorithms; and integer variables lead to combinatorial growth of the solution space. We investigate these complexity factors by employing gas flow models for network optimization. Because the nonconvex dynamic model is intractable for network optimization, we use the SOC dynamic model as the reference.

**Table 1:** Mathematical Formulation of Gas Flow Models

model	variables	constraints	penalty term
nonconvex dynamic	*p*_*mt*_, *p*_*nt*_, ϕ_*mnt*_, *ϕ*_*mnt*_^in^, *ϕ*_*mnt*_^out^	(9), (10), (12), (13)	
SOC dynamic	*p*_*mt*_, *p*_*nt*_, *p*_*mnt*_^min^, *p*_*mnt*_^max^, *ϕ*_*mnt*_, *ϕ*_*mnt*_^in^, *ϕ*_*mnt*_^out^, *y*_*mnt*_, γ_*mnt*_	(9), (12), (13), (14), (16), (18)	(17)
SOC static	*β*_*mt*_, *β*_*nt*_, *β*_*mnt*_, *ϕ*_*mnt*_, *ϕ*_*mnt*_^in^, *ϕ*_*mnt*_^out^, *y*_*mnt*_, γ_*mnt*_	(13), (19), (20), (22), (23), (24)	(17)
linear static	*p*_*mt*_/*β*_*mt*_, *p*_*nt*_/*β*_*nt*_, *ϕ*_*mnt*_, *ϕ*_*mnt*_^in^, *ϕ*_*mnt*_^out^	(13), (19), (12)/(24), (25), (26)	

**Table 2:** Complexity Factors of Gas Flow Models

model	nonconvex	time-linking	nonlinear	mixed-integer
nonconvex dynamic	×	×	×	
SOC dynamic		×	×	×
SOC static			×	×
linear static				

Similar to the linear static model for pipes, we model other network components, namely, compressors and regulating valves, using alternative pressure variables *p* and β as detailed in the literature.^9,30^ These alternative formulations ensure compatibility with the SOC dynamic and SOC static gas flow models, which use *p* and β variables, respectively.

## 3 Accuracy Metrics

Four metrics assess the steady-state and the transient accuracy of the gas flow models of Section 2, with the first two investigated for single pipes and the last two investigated for gas networks.

### 3.1 Transfer Capacity

Transfer capacity is the maximum mass flow in a pipe at the steady state and is computed as the minimum between the cross-section capacity, , and the pressure-induced bound, , set by eqs 5 and 7, respectively. Using the constraints in Table 3, the discrete models capture the exact transfer capacity.

**Table 3:** Constraints Capturing Transfer Capacity in Discrete Gas Flow Models

model	cross-section capacity	pressure-induced bound
nonconvex dynamic	(13)	(10), (12)
SOC dynamic	(13)	(12), (14)
SOC static	(13)	(20), (24)
linear static	(13)	(26)^a^

a Exact only with one-segment discretization.

### 3.2 Transient Pressure Error

For assessing the transient response of the gas flow models in a single pipe, the pressure at the pipe inlet is fixed, a step load is applied at the pipe outlet, and the transient response is benchmarked against a reference model, i.e., the SOC dynamic model with fine spatial discretization. This is achieved by computing the mean absolute error of the outlet pressure
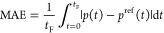
27where *p*(*t*) and *p*^ref^(*t*) are the outlet pressures in the test and reference models, respectively; *t*_*F*_ is a time instant when *p*^ref^(*t*) has reached the steady state. To eliminate the influence of the step load’s amplitude, the pressure values are scaled such that *p*^ref^(0) = 1 and lim_*t*→∞_*p*^ref^(*t*) = 0.

### 3.3 Pressure Drop Error

The pressure drop error is the deviation from the Weymouth equation (eq 10)
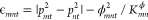
28which is equal to the relaxation gap in the SOC models.

In the SOC dynamic model, eqs 12 and 14 bound the error as
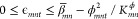
29

In the SOC static model, eqs 20a and 22a,22b yield
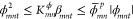
30

Subtracting *ϕ*_*mnt*_^2^ and dividing by K_*mn*_^ϕ^ gives
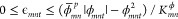
31

In the linear static model
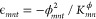
32which is bounded by the cross-section capacity, eq 5, as
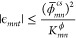
33

**Figure 2:**
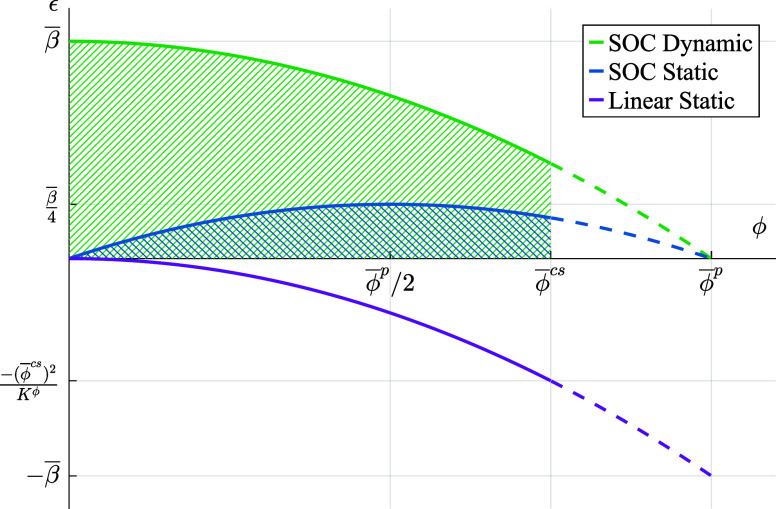
Pressure drop error of gas flow models for varying mass flow rates.

The error range of the pressure drop for the three models is depicted in Figure 2 for varying mass flow rates ϕ. Setting ϕ determines the error of the linear static model but yields a bound on only the error of the SOC models. The hatched areas indicate that the actual error in the SOC models lies between the bound and zero, depending on the relaxation gap. The maximum error of the SOC static model is 1/4 of that of the SOC dynamic model, owing to eq 22a,22b. The error sign shows that the SOC models may overestimate the pressure drop, while the linear static model underestimates the pressure drop. The error magnitude signifies the distance from a physically feasible flow.

If the linearization parameters and penalty coefficients in eq 17 are appropriately set, the errors of the SOC models are significantly smaller than the identified bounds. Hence, we focus on the error bound of the linear static model, eq 33, and express it as
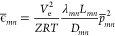
34which is proportional to the pipe’s length–diameter ratio. For typical natural gas and pipe properties, such as those reported in Section 5, the error  if the length–diameter ratio *L*/*D* ≪ 51000. In the case of hydrogen, the molecular weight, viscosity, and volumetric energy density are lower; as a result, the pressure drop and hence the error  are much lower for equal flow velocity compared with natural gas, but up to 20% higher for equal energy rates.^36^

### 3.4 Gas Supply Cost

A typical objective of gas network operations is to minimize the total cost of the gas supply
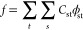
35where *C*_st_ and ϕ_st_ are, respectively, the specific cost and mass flow of gas supplier *s* at time *t*.

Gas supply cost can be optimized by leveraging the linepack flexibility of pipes, which serve as network storage. Static models do not capture linepack flexibility and therefore may yield suboptimal solutions. Hence, the gas supply cost is used for benchmarking static models against dynamic models.

## 4 Solution Procedure for Network Optimization

Using the SOC dynamic model for the gas flow constraints in eq 1 results in a mixed-integer SOC program, which can be solved via commercial solvers such as Gurobi,^37^ MOSEK,^38^ and CPLEX.^39^ Yet, the complexity factors indicated in Table 2 preclude tractability for horizons beyond 168 time steps or networks with more than 18 pipes. Therefore, we developed an efficient solution procedure, Algorithm, for network optimization. The two-stage optimization structure not only serves computational efficiency but also enhances relaxation quality by constructing relaxation penalty terms. The solution procedure builds on pipe aggregation, pipe classification, and fixing flow directions. These techniques, respectively, address the computational complexities arising from problem size, nonlinearity, and integer variables and are detailed in the following.
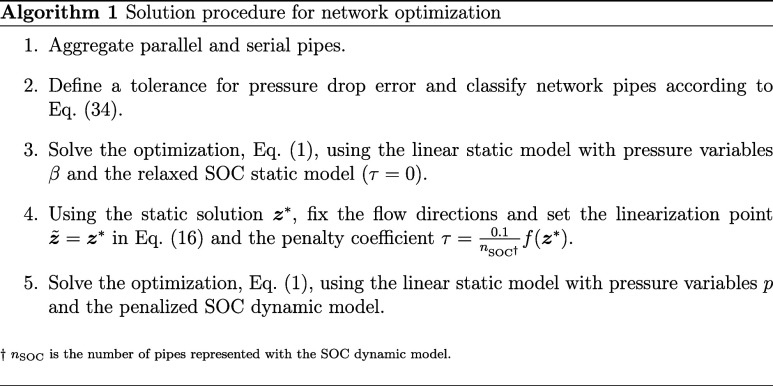


### 4.1 Pipe Aggregation

**Figure 3:**
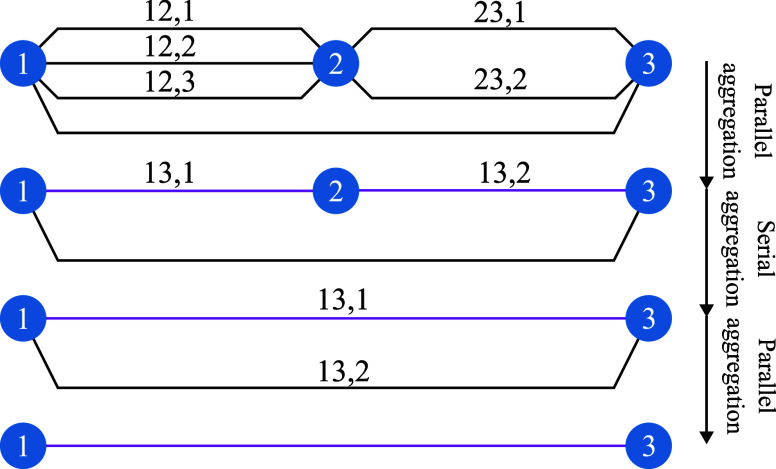
Recursive pipe aggregation. The labeled pipes are aggregated to purple-colored pipes.

To reduce the problem size, parallel and serial pipes are recursively aggregated into equivalent pipes, as illustrated in Figure 3, until no further pipes can be aggregated.

When aggregating pipes, the total linepack must be preserved to ensure identical transient behavior. Hence, eq 9b requires
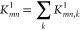
36where *k* enumerates parallel or serial pipes.

For computing the equivalent *K*_*mn*_^ϕ^, we use the Weymouth equation, eq 10, and the pressure and flow relations in parallel and serial configurations. Specifically for parallel pipes, the pressure drop is identical for individual pipes, and the total flow equals the sum of flows in individual pipes, resulting in
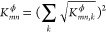
37

For serial pipes, the pressure is consistent in adjacent pipes, and the flows in individual pipes are equal under the steady state, resulting in
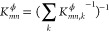
38

Having computed *K*_*mn*_^l^ and *K*_*mn*_^ϕ^, we can compute equivalent pipe properties *L*_*mn*_, *D*_*mn*_, and λ_*mn*_ using eq 2.2 and an additional assumption to obtain a fully determined equation system. Assuming equal length, *L*_*mn*_ = *L*_*mn*,*k*_, for parallel pipes and equal diameter, *D*_*mn*_ = *D*_*mn*,*k*_, for serial pipes, we summarize the equivalent pipe properties in Table 4.

**Table 4:** Properties of Equivalent Pipes Resulting from Pipe Aggregation

property	parallel pipes	serial pipes
*L*_*mn*_	*L*_*mn*,*k*_	∑_*k*_*L*_*mn*,*k*_
*D*_*mn*_	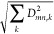	*D*_*mn*,*k*_
λ_*mn*_	*D*_*mn*_^5^[∑_k_(*D*_*mn*,*k*_^5^/λ_*mn*,*k*_)^1/2^]^−2^	∑_*k*_(λ_*mn*,*k*_*L*_*mn*,*k*_)/*L*_*mn*_

### 4.2 Pipe Classification

**Figure 4:**
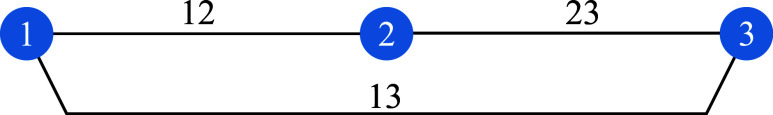
Pipe classification example. Pipes are labeled using the start and end nodes. Pipes 12 and 23 meet the tolerance for the pressure drop error and qualify for the linear static model, which enforces equal pressure at nodes 1 and 3. However, using the SOC models for pipe 13 would imply zero flow, and, therefore, the linear static model is also used for pipe 13. While the linear static model allows nonzero flow in pipe 13, the associated pressure drop error may exceed the predefined tolerance. We accept this possibility to favor a higher accuracy of flow capacities, especially because computational experiments show that pressure drop errors are typically much smaller than the respective bounds.

We select a tolerance for the pressure drop error, which could be based on the allowable incidental exceedance of normal operating pressure ranges prescribed in pipeline safety regulations.^40^ Given this tolerance, we use the error bound in eq 34 to classify network pipes. Specifically, we use the linear static model for pipes meeting the tolerance (i.e., ) and for the pipes directly connecting their end points as illustrated in Figure 4. The SOC dynamic model is used for the remaining pipes, which tend to have the highest length–diameter ratios, according to eq 34. Such a classification reduces the computational burden while maintaining the pressure drop error and transient pressure error at acceptable levels. Our approach improves on the *short pipe model* in the literature^30^ via the inclusion of pressure-induced flow bounds, eq 26, and proposing a systematic procedure for classifying network pipes considering accuracy requirements.

### 4.3 Fixing Flow Directions

We tackle the computational complexity of the SOC dynamic model stemming from the integer variables indicating the flow directions by fixing their values according to the solution of the SOC static model optimization. Indeed, fixing the integer variables predetermines flow directions in dynamic flow optimization; however, it does not mandate unidirectional flows. Indeed, the flow directions may change from one time step to another to ensure the economic gains of bidirectional flows.^12,41^ Notably, other researchers have also adopted the concept of fixing flow directions, but via other simplified optimization forms.^9,11,17,18,31^ Furthermore, we extend this concept to fix flow directions in compressors and regulating valves.

## 5 Results

The results are organized in three parts. Section 5.1 establishes the SOC dynamic model with fine spatiotemporal resolution as the benchmark via a transient validation on a literature test network. Compared to the benchmark, Section 5.2 demonstrates the accuracy of simplified models with one-segment spatial discretization for single pipes. Section 5.3 assesses the accuracy and computational performance of the proposed solution procedure for gas network optimization with an hourly resolution, aligning with the temporal resolution in energy system expansion planning studies.^29^ We set *V*_e_ = 15.24 m/s^42^ and use Gurobi^37^ for solving the optimizations.

### 5.1 Model Validation on a Literature Test Network

**Figure 5:**
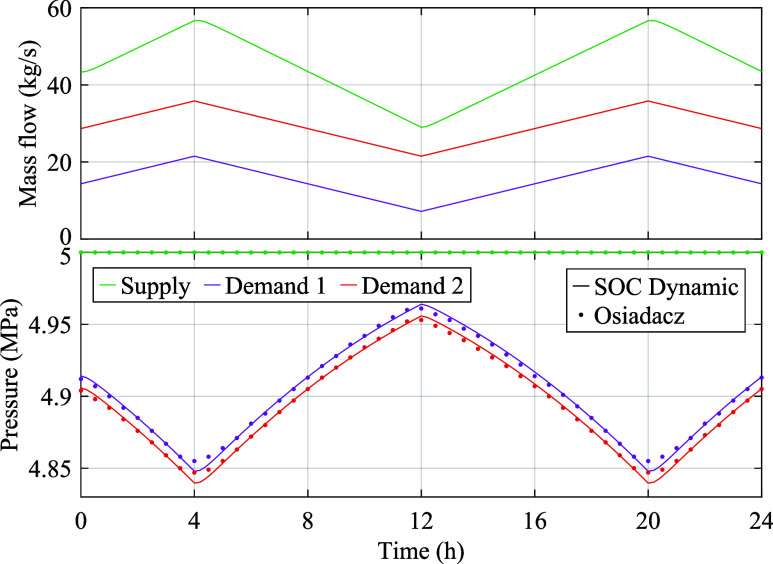
Mass flow and pressure evolution in Network 0 under constant supply pressure. Pressure values are validated against literature transient simulations.^43^

We simulate a three-node network (Network 0),^43^ detailed in the Supporting Information, using the SOC dynamic model with eight-segment discretization and Δ*t* = 720 *s*. The demands are varied, as depicted in the upper panel of Figure 5. The lower panel shows the pressure evolution at the demand nodes under constant supply pressure, validated against the transient simulation conducted by Osiadacz.^43^ The root-mean-square and maximum errors of the pressures are below 0.05 and 0.16%, respectively. This validation demonstrates the accuracy of the SOC dynamic model, which serves as a benchmark for subsequent model comparisons.

**Figure 6:**
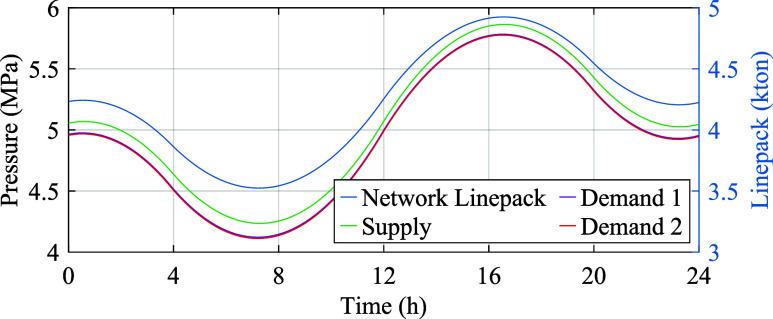
Pressure and linepack evolution in Network 0 under constant supply mass flow.

Linepack flexibility can buffer the supply against demand fluctuations. This is illustrated in Figure 6 for Network 0 under variable supply pressure. Above-average demands coincide with pressure reduction, indicating the contribution of the linepack in serving the demands. Conversely, during below-average demands, the pipes are *packed*, maintaining a constant supply mass flow.

### 5.2 Accuracy Assessment for Single Pipes

**Figure 7:**
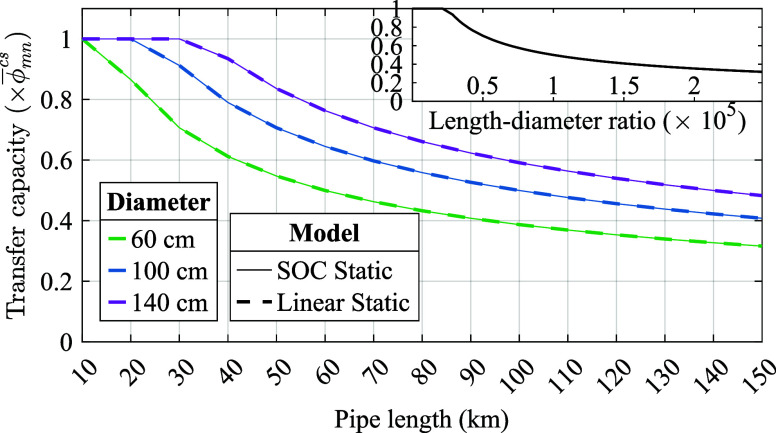
Impact of the pipe length and diameter on transfer capacity normalized by cross-section capacity .

We assess the accuracy of flow models for single pipes by using transfer capacity and transient pressure. The nominal parameter values are  m/s^44^ and λ = 0.01^45^ based on measured natural gas properties. Figure 7 shows the dependence of transfer capacity on pipe properties for one-segment static models, both aligning with the benchmark model as indicated in Section 3.1. The transfer capacity is equal to the cross-section capacity for short pipes with large diameters. Conversely, for pipes with a length–diameter ratio above 25000 in Figure 7, the transfer capacity is restricted by the permissible pressure drop and exhibits an inverse relationship with this ratio, in accordance with eq 8. Therefore, the linear static model extends the steady-state accuracy of the *short pipe model*,^30^ which neglects pressure-induced flow bounds, to pipes with higher length–diameter ratios. The pipe transfer capacity is analogous to power line loadability.^46^ For short lines, it is determined by the thermal rating, which depends on the line’s cross-sectional area. Meanwhile, for long lines, it is dictated by the permissible voltage drop and the stability margin, which depend on the line’s length.

**Figure 8:**
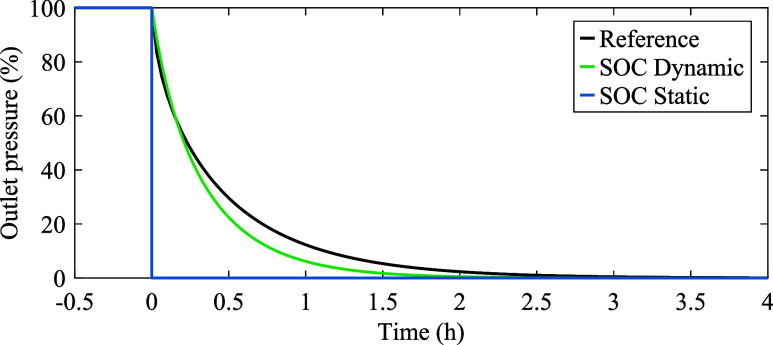
Pressure evolution in a 100 km pipe with a diameter of 60 cm for three gas flow models in response to a step load of 20% of the cross-section capacity, using a time step of Δ*t* = 120 s.

We assess the transient response of the gas flow models under a step load with an amplitude equal to 20% of the cross-section capacity. Figure 8 shows the outlet pressure of a 100 km pipe with a diameter of 60 cm for three gas flow models. The reference model is an SOC dynamic model with eight-segment discretization, and the test models are SOC static and SOC dynamic models with one-segment discretization. The reference model’s finer discretization qualifies as a benchmark for the test models. The one-segment SOC dynamic model predicts a faster convergence of the pressure to the final value compared to the reference model. As expected, the SOC static model fails to capture the transient pressure evolution and assumes an immediate pressure drop. Notably, the SOC relaxation gaps are zero in this experiment.

**Figure 9:**
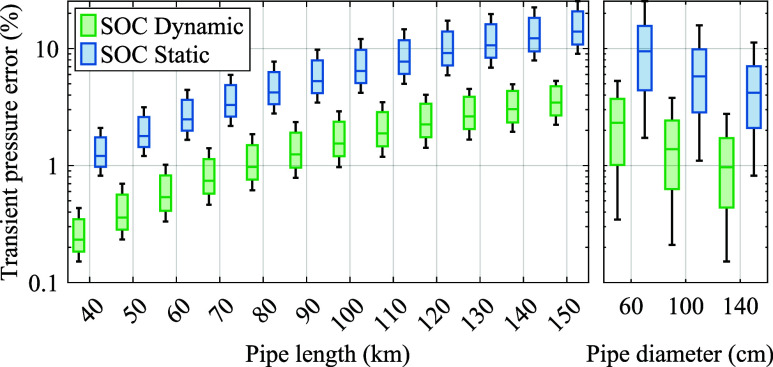
Transient pressure error of one-segment SOC models.

We repeat the transient experiment, exploring variations in  and λ by ±11 and ±10%, respectively. Additionally, we investigate the effects of varying pipe length and diameter, as illustrated in Figure 9, on the transient pressure drop error (eq 27). The errors quadratically increase with the pipe length, which equals the discretization length here, aligning with the second-order accuracy of the midpoint method for spatial discretization.^35^ The errors are higher for lower diameters. In terms of accuracy, the SOC dynamic model surpasses the SOC static model by 75–82%, with the absolute discrepancy being higher for pipes with longer lengths and lower diameters. This underscores the rationale behind pipe classification according to eq 34, which integrates the length–diameter ratio. As the transient error due to spatial discretization is small compared to the error from temporal discretization,^35^ the SOC dynamic model with one-segment discretization, i.e., serial pipe aggregation, is sufficiently accurate for hourly resolved studies.

### 5.3 Accuracy and Computational Performance of Network Optimization

We formulate an optimization problem, eq 1, minimizing the gas supply cost, eq 35. The solution employs the procedure proposed in Section 4 on a computation node equipped with two 64-core AMD EPYC 7742 processors and up to 2 TB RAM and with a 1 h cap on solution time. While maintaining an hourly time step^29^ (Δ*t* = 3600 s), we assess temporal scalability by conducting optimizations on various time horizons, including a day, a week, a month, a quarter, and a year (number of time steps *n*_*t*_ ∈ {24, 168, 720, 2184, 8760}). Motivated by the accuracy assessments in Section 5.2, the discretization length of the pipes is selected equal to the pipe length.

**Figure 10:**
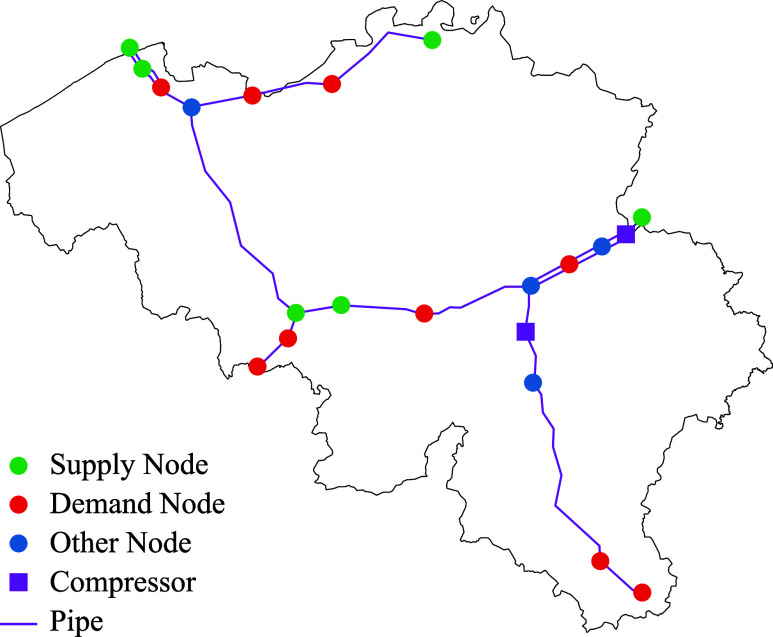
Belgian natural gas network used in supply cost minimization analyses.

Three natural gas networks of various sizes are considered to assess spatial scalability: the Belgian network^47^ (Network 1), a synthetic network^48^ (Network 2), and the Swiss network^9^ (Network 3). For demand time series, we use historical Belgian gas consumption^49^ in Networks 1 and 2 and use measured flows from the Swiss network in Network 3. The network data are provided in the Supporting Information for reproducibility. We present overarching results for the three networks, with more comprehensive results for Network 1, which are shown in Figure 10.

Gas supply costs vary among suppliers. We cap hourly supply from the cheapest suppliers to match the average daily demand. As a result, the cheapest suppliers can serve the entire demand with a constant intraday supply equal to their caps, subjected to sufficient flexibility in the network for buffering intraday demand fluctuations. The corresponding supply cost, therefore, serves as a lower bound on the optimal supply cost and is used for benchmarking the supply cost obtained from various models. The specific costs of the gas supply are scaled according to historical monthly TTF prices.^50^

We assess the accuracy and computational performance of the solution procedure across variations in the algorithm, with respect to the tolerance for the pressure drop error (step 2), the penalty terms (steps 3 and 4), and fixing flow directions (steps 3 and 4).

**Figure 11:**
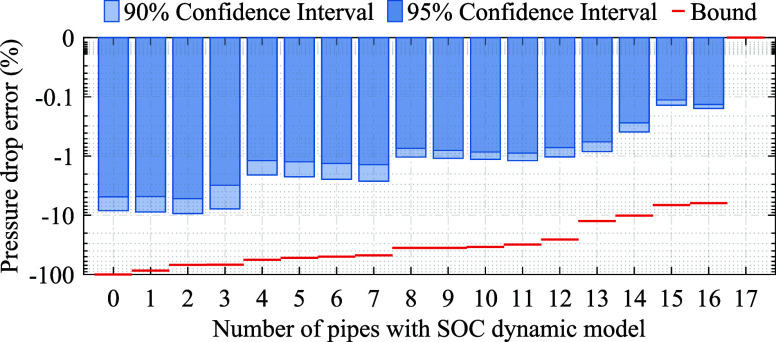
Pressure drop error relative to the maximum network pressure assessed for the linear static model in Network 1. The tolerance for the pressure drop error is varied to obtain 18 pipe classifications. In each classification, the number of samples equals the number of time steps times the number of pipes with the linear static model, i.e., (24 + 168 + 720 + 2184 + 8760) × (17 – *n*_SOC_).

We vary the tolerance for the pressure drop error to obtain various pipe classifications according to step 2 of the Algorithm. The errors are normalized by maximum network pressure  and depicted in Figure 11. Tightening the tolerance requires capturing a higher number of pipes with the SOC dynamic model, which results in a tighter error bound, eq 34. Notably, the reduction of error bounds correlates with reduced 90th and 95th percentiles of pressure drop errors across various pipes and time horizons; although, the percentiles are consistently smaller than 13.6% of the error bounds, which is because pipes are rarely operated close to their cross-section capacities in Network 1.

Table 5 compares the pressure drop error of the gas flow models. The 95th percentile of the error for the linear static model is below 8.4% of the bound, eq 34, for Networks 1 and 2, and it is equal to the bound for Network 3. The 95th percentiles of the errors are up to 36.3% for the relaxed SOC dynamic model (with a zero penalty coefficient). In contrast, these percentiles are below 0.03% for the penalized SOC dynamic model. For both SOC dynamic models, the errors are notably smaller than the bounds derived from single pipeline analysis. This is attributable to the network requirements that prohibit the concurrent overestimation of the pressure drop in network pipes. Therefore, the modeling requirements of an independently considered pipeline section are more stringent compared with the case when a pipeline section is part of a network.^51^ Overall, capturing more pipes with the penalized SOC dynamic model tightens the negative pressure drop error related to the linear static model, while the positive pressure error remains negligible, owing to the penalty terms. Specifically, capturing a minimum of 14 and 6 pipes with the penalized SOC dynamic model limits the 95th percentile of the error magnitude to 0.4% in Networks 1 and 2, respectively.

**Table 5:** Pressure Drop Error Expressed as % of the Maximum Network Pressure for the Linear Static and the SOC Dynamic Gas Flow Models^a^

Network	Network 1	Network 2	Network 3
linear static model (bound)	–100.000	–60.938	–86.827
linear static model (P95)	–8.356	–3.924	–86.667
linear static model (P90)	–4.869	–1.988	–86.667
SOC dynamic model (bound)	100.000	60.938	86.827
SOC dynamic model—relaxed (P95)	35.843	26.068	36.276
SOC dynamic model—relaxed (P90)	27.832	22.732	32.114
SOC dynamic model—penalized (P95)	0.025	0.005	0.001
SOC dynamic model—penalized (P90)	0.000	0.003	0.000

a “Linear static model” refers to the case in which all pipes are captured with the linear static model. “SOC dynamic model” refers to the case in which 17, 14, and 5 pipes are captured with the SOC dynamic model in the three networks, respectively, and the remaining pipes are captured with the linear static model.

Table 6 shows that the average gas supply cost from the SOC dynamic model is merely 0.01–0.06% higher than the cheapest supply cost. This reveals that the linepack flexibility in the networks effectively accommodates the intraday demand fluctuations. The linear static model, by disregarding linepack flexibility, incurs up to 3.1% additional cost. The penalized SOC solution is practically feasible, as the relaxation gaps are closed. Therefore, the optimal cost is expected to lie between the costs of the relaxed and penalized solutions. The length of this interval is consistently below 0.1%, indicating the high quality of the penalized solution. As the solution time of the penalized model is only 16–65% higher, we use the penalized model hereafter.

**Table 6:** Gas Supply Cost Expressed as % of the Cheapest Supply Cost for the Linear Static and the SOC Dynamic Gas Flow Models^a^

Network	Network 1	Network 2	Network 3
linear static model	101.755	102.581	100.518
SOC dynamic model—relaxed	100.027	100.010	100.008
SOC dynamic model—penalized	100.057	100.010	100.060

a “Linear static model” refers to the case in which all pipes are captured with the linear static model. “SOC dynamic model” refers to the case in which 17, 14, and 5 pipes are captured with the SOC dynamic model in the three networks, respectively, and the remaining pipes are captured with the linear static model.

**Figure 12:**
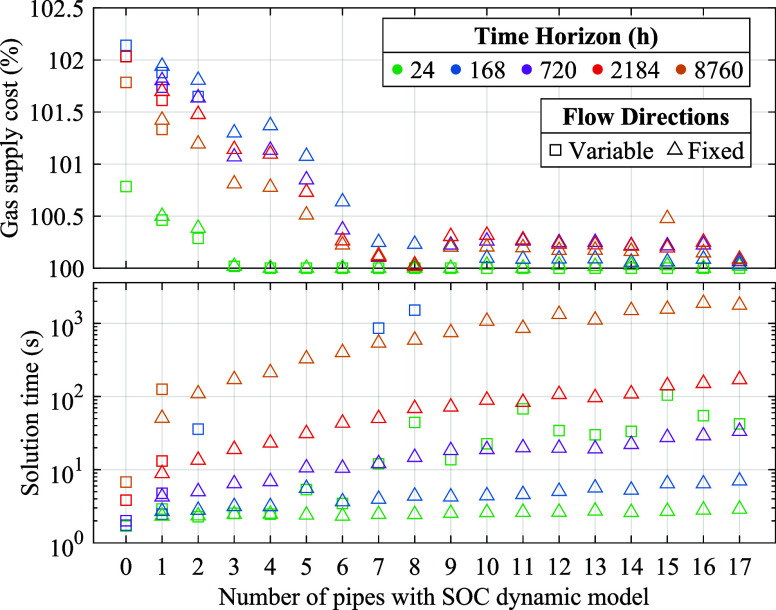
Impact of the gas flow model on the gas supply cost (expressed as % of the cheapest supply cost) and solution time for Network 1.

Figure 12 illustrates the impact of modeling choices related to flow direction variables and the number of pipes represented with the SOC dynamic model on the gas supply cost and the solution time for various time horizons in Network 1. A cost saving of 0.8–2.0% is realized if 3–7 pipes are captured with the SOC dynamic model. Remarkably, the computational requirements increase hyperlinearly with the number of pipes with the SOC dynamic model (*n*_SOC_) and the number of time steps (*n*_*t*_). The experiments on the networks predict a solution time of  and maximum memory usage of , where *a* ∈ [1.30,1.67], *b* ∈ [1.08,1.74], *c* ∈ [1.33,1.59], and *d* ∈ [1.85,1.97]. This highlights the computational gain that the proposed solution procedure achieves through pipe aggregation and selectively by using the SOC dynamic model.

We assess the implications of fixing flow directions by excluding steps 3 and 4 of the solution procedure in the Algorithm. Figure 12 demonstrates that fixing flow directions significantly reduces the solution time while incurring minor cost suboptimality in Network 1. Table 7 quantifies the change in computational and accuracy metrics due to fixed flow directions in the three networks. The success rate denotes the ratio of successful optimizations given the allocated computational resources. As a result of obtaining a continuous model, the success rate is enhanced by a minimum of 96%. For successful optimizations, the solution time decreases by 20–69% on average and up to 3 orders of magnitude for the cases with many direction variables. The solution of the SOC dynamic model with fixed directions takes on average 21–37% of the total solution time, and the rest is spent in solving the static model with variable directions, implying the greater computational complexity posed by integer variables denoting flow directions compared to the time-linking constraints in the SOC dynamic model. Fixing flow directions restricts the feasible region of the SOC dynamic model and, as a result, incurs a suboptimality with a 95th percentile up to 0.3% but does not render the model infeasible in any of the instances.

**Table 7:** Impact of Fixing Flow Directions on Computational Performance and Gas Supply Cost

Network	Network 1	Network 2	Network 3
success rate increase (%)	210.345	102.222	96.000
avg. solution time decrease (%)	62.236	20.472	68.781
P90 suboptimality (%)	0.163	0.019	0.270
P95 suboptimality (%)	0.238	0.204	0.313

## 6 Conclusions

This paper proposes a computationally efficient procedure for solving network optimizations based on three discrete gas flow models, namely, the SOC dynamic model, the SOC static model, and the linear static model. These models capture the dependence of transfer capacity on the pipe length due to pressure bounds. The computational experiments on single pipes and gas networks support the following key findings:

1. The SOC static model assumes immediate pressure adjustment in response to a step load, whereas the SOC dynamic model with a one-segment discretization exhibits a minor overestimation of the dynamic speed, resulting in a reduction of the transient error by an average of 77%. Specifically, the SOC dynamic model with one-segment discretization is sufficiently accurate for hourly resolved studies such as energy system expansion planning.
2. The omission of linepack flexibility in static models prevents its contribution to buffering supply against demand fluctuations. Consequently, the case studies highlight an overestimation of the gas supply cost by up to 3.1%. While this increase holds significance for daily operations, its relevance might diminish when weighed against longer-term uncertainties, such as gas price forecasts.
3. We use the solution from the SOC static model to fix the integer variables in the SOC dynamic model and construct relaxation penalty terms. As a result, the solution time is reduced by 20.5–68.8% on average, and the 95th percentile of the relaxation gaps is tightened to 0.03%, while the incurred suboptimality remains below 0.3% in 95% of the instances.
4. Computational needs increase hyperlinearly with the number of pipes with the SOC dynamic model. The proposed solution procedure significantly enhances the computational efficiency through pipe aggregation and selectively using the SOC dynamic model. As a result, an accurate gas supply cost is achieved for representing merely 3–29% of the pipes with the SOC dynamic model. This is particularly pertinent to achieve tractability in optimizing large networks over long horizons.

The proposed solution procedure demonstrated scalability to 8760 time steps, which is the benchmark in energy system expansion planning.^29^ Yet, if the performance should be enhanced when including investment decisions, the employed concept of fixing flow directions can be readily extended to fixing investment decisions. Furthermore, temporal aggregation techniques^52^ can reduce problem size without sacrificing essential details. Future work can explore replacing the linear static model or SOC dynamic model with a linear relaxation^12^ for enhanced accuracy or computational efficiency.

## Supporting Information Available

Data of the gas networks (XLSX)
